# Lipid reprogramming induced by the NNMT-ABCA1 axis enhanced membrane fluidity to promote endometrial cancer progression

**DOI:** 10.18632/aging.205142

**Published:** 2023-10-25

**Authors:** Qirong Wen, Xiaohui Xie, Caiyuan Chen, Bolun Wen, Yaqiong Liu, Jie Zhou, Xiaobin Lin, Han Jin, Kun Shi

**Affiliations:** 1 Department of Gynecology and Obstetrics, Guangzhou Women and Children’s Medical Center, Guangzhou Medical University, Guangzhou, China; 2 Department of Breast Surgery and General Surgery, Guangzhou Women and Children’s Medical Center, Guangzhou Medical University, Guangzhou, China; 3 Prenatal Diagnosis Center, Guangzhou Women and Children’s Medical Center, Guangzhou Medical University, Guangzhou, China; 4 Department of Obstetrics and Gynecology, Guangzhou Medical University, Guangzhou, China

**Keywords:** cholesterol metabolism, epithelial-mesenchymal transition, methylation endometrial cancer

## Abstract

Elucidating the mechanism for the high metastasis capacity of Endometrial cancer (EC) is crucial to improve treatment outcomes of EC. We have recently reported that nicotinamide N-methyltransferase (NNMT) is overexpressed in EC, especially in EC, and predicts poor survival of chemotherapy patients. Here, we aimed to determine the function and mechanism of NNMT on metastasis of EC. Additionally, analysis of public datasets indicated that NNMT is involved in cholesterol metabolism. *In vitro*, NNMT overexpression promoted migration and invasion of EC by reducing cholesterol levels in the cytoplasm and cell membrane. Mechanistically, NNMT activated ABCA1 expression, leading to cholesterol efflux and membrane fluidity enhancement, thereby promoting EC's epithelial-mesenchymal transition (EMT). *In vivo*, the metastasis capacity of EC was weakened by targeting NNMT. Our findings suggest a new molecular mechanism involving NNMT in metastasis, poor survival of EC mediated by PP2A and affecting cholesterol metabolism.

## INTRODUCTION

Patients’ current and future health might be affected by their body mass index (BMI), which is regarded as one of the top five global causes of mortality for females [1]. Endometrial cancer (EC) is the kind of cancer that is most strongly connected with obesity. Obesity has been recognised as a very substantial risk factor for EC in postmenopausal women [2], and obesity is the type of cancer most strongly related to obesity. EC is the sixth most prevalent type of cancer in females and the second most common type of gynaecological malignancy. In the United States in 2021, roughly 66,570 newly diagnosed cases of EC and 12,940 fatalities were reported as a result of the disease [3]. In recent years, there has been a discernible rise in the number of cases of EC in China. From 2012 to 2015, the age-standardised incidence of EC was 63.4 cases per 100,000 people, the death rate was 21.8 cases per 100,000 people, and the relative 5-year survival rate was 72.8% [4]. The study has been centred on tumor lipid metabolism due to the fact that patients with EC also experience the symptoms associated with being overweight and having diabetes. On the other hand, the mechanism behind EC’s lipid metabolism is not yet fully understood.

Cancer may be identified by a number of telltale signs, including the disruption of cellular metabolism and the activation of invasion and metastasis [2]. Cancer metabolism depends on modifications in critical metabolic pathways and significantly impacts the expression of oncogenes and tumor suppressor genes. These genes are essential in controlling cancer cell proliferation, apoptosis, autophagy, and metastasis. Nicotinamide N-methyltransferase, also known as NNMT, is a cytosolic enzyme that catalyses the transfer of the methyl units from S-adenosyl-l-methionine, also known as SAM, to nicotinamide, also known as NAM. This results in the production of the stable metabolic product 1-methyl nicotinamide, also known as 1-MNA. Because of this, NNMT expression is responsible for controlling the methylation potential of cancer cells. This, in turn, causes an altered epigenetic state characterised by hypomethylated histones and other cancer-related proteins, such as tumor suppressor PP2A [3]. Based on this fundamental function, the overexpression of NNMT in various types of cancer, including EC, impairs proliferation, apoptosis, autophagy, nutrient metabolism, and metastasis, which in turn affects the treatment and survival of patients with liver, prostate, gastric, and pancreatic cancer.

Cholesterol is an essential biomolecule that plays a vital role in various physiological and pathological activities. Alterations in the free cholesterol level and phospholipid species of the plasma membrane are responsible for modulating the signalling of numerous receptors [1]. The ATP-binding cassette transporter A1 (ABCA1) is a transmembrane protein extensively expressed in many tissues. In these tissues, it is thought to perform various activities. ABCA1 is only one of numerous proteins that are involved in cholesterol homeostasis. Its most well-studied function is the efflux of intracellular free cholesterol and phospholipids across the plasma membrane, where they combine with apolipoproteins, most notably apolipoprotein A-I (ApoA-I), to form nascent high-density lipoprotein particles (HDLs). This is the first step in reverse cholesterol transport (RCT) [5]. In addition to its role in the production of high-density lipoprotein cholesterol (HDL-C), the ABCA1 gene controls the cholesterol and phospholipid content of the plasma membrane. It also has a role in producing microparticles and, by extension, cell signalling. Due to the fact that altered cholesterol homeostasis can have an effect on a wide variety of organs and is implicated in the pathophysiology of a large number of disorders, ABCA1 is an essential biomolecule.

PGC-1α (regulatory gene structure-1α) and ERRα (mimetic factor nuclear receptor α) are two gene regulators closely related to the regulation of energy metabolism and cell function. PGC-1α is a transcriptional coactivator that regulates the expression of many genes, especially those related to cellular energy metabolism and mitochondrial function. PGC-1α regulates cellular energy metabolism and oxidative stress by working with transcription factors to promote mitochondrial biosynthesis and enhanced function of the mitochondrial respiratory chain. ERRα is closely related to PGC-1α, and they can interact and co-regulate the expression of a range of genes that affect the aerobic metabolism and mitochondrial function of cells. ERRα mainly regulates fatty acid oxidation and glycolytic pathways to maintain the energy balance of cells [6–10]. In patients with endometrial cancer, the use of PGC-1α and ERRα has attracted widespread attention. Some studies have shown that increasing the expression of PGC-1α and ERRα can inhibit the proliferation and invasion capacity of endometrial cancer cells and promote apoptosis. This means that the application of PGC-1α and ERRα may have the potential to inhibit endometrial cancer tumor growth and metastasis. In addition, the use of PGC-1α and ERRα may also improve treatment response and prognosis by improving cellular energy metabolism and reducing oxidative stress [11–14].

There is a connection between the EMT program and the lipid modification of the cell membrane [15]. The fatty acyl moieties of membrane phospholipids display a substantial variability in chain length as well as varying degrees of saturation. The fatty acyl moieties determine these biophysical aspects of membranes, including their fluidity, curvature, and subdomain architecture. Phosphatidylcholine (PC) is the most abundant phospholipid in mammalian cell membranes and subcellular organelles, accounting for 40–50% of the total phospholipids [16, 17]. Glycerophospholipids (GPs) are the primary structural phospholipids found in mammalian membranes. [Note: GPs are also known as glycerophospholipids]. To maintain oncogenic activity in a wide range of malignancies, PC’s saturability affects the plasma membrane of tumor cells [16, 18]. Researchers Lin et al. demonstrated that the length of the membrane’s fatty acid chain might influence the plasma membrane’s fluidity and the invasion of liver cancer [19]. There is still a lot of mystery around the functions lipid metabolism plays in the membrane in EC. Interestingly, reports have indicated that NNMT and ABCA1 are implicated in the lipid remodelling signalling pathway.

Research is still needed to fully understand how NNMT plays a role in lipid metabolism and how this process influences the metastasis and invasion of malignant cells. We predicted that NNMT-ABCA1 signalling, which regulates lipid metabolism, greatly influences membrane function to increase EC invasion and metastasis. This was based on the fact that lipid metabolism is regulated by NNMT-ABCA1. This study highlights the interaction between NNMT and ABCA1 as well as their coregulation of FA metabolism to enhance invasion and metastasis in EC. Our group carried out the study.

## RESULTS

### Bioinformatics analysis revealed that NNMT promotes ABCA1 expression to participate in EC progression

**Figure 1:**
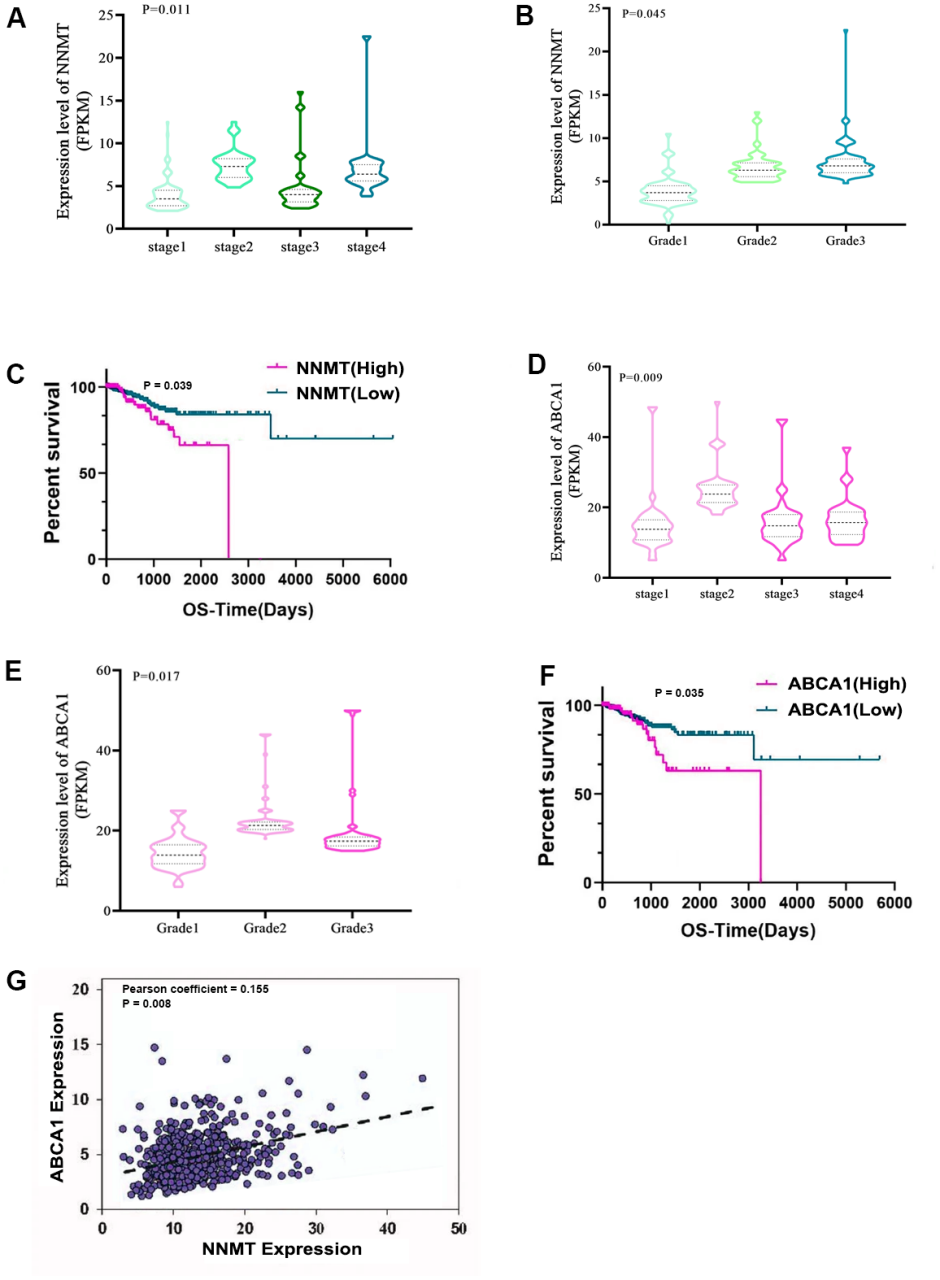
**Bioinformatics analyses show that NNMT stimulates ABCA1 transcription, contributing to EC development.** Results from the TCGA database (norm = 23, extreme = 543) are displayed. Variations in NNMT expression may be seen (**A**) throughout all FIGO stages (**B**) and pathological grades. (**C**) The quartile-based correlation between NNMT and OS is displayed (Low: 1st quartile distribution; Median: 2nd-3rd quartile distribution; High: 4th quartile distribution). ABCA1 expression varies among FIGO stages (**D**) and pathogenic subtypes (**E**). Shown below is the link between ABCA1 and OS (**F**). (**G**) The relationship between NNMT and ABCA1 expression in EC tissue.

The expression and clinicopathological data of NNMT and ABCA1 were initially analysed in 543 EC patients and 23 normal samples from the TCGA RNA-seq collection to further understand their potential role in EC. High NNMT expression was substantially linked to a later stage of EC (P < 0.05; Figure 1A) but not to a higher pathological grade (P > 0.05; Figure 1B). Finally, Figure 1C shows that patients with EC who expressed high levels of NNMT had a significantly shorter overall survival (OS) than those who expressed low levels of NNMT. Similarly, higher stages and grades of EC were linked considerably with greater ABCA1 expression (P < 0.05; Figure 1D–1F). The expression of NNMT was positively correlated with that of ABCA1 (Pearson coefficient = 0.197; P < 0.001; Figure 1G), which accorded with our hypotheses.

### NNMT promotes cell migration and invasion of EC cells

We initiated our investigation into the role of NNMT in EC metastasis *in vitro* by screening EC cell lines for NNMT expression in our laboratory. Low NNMT expression is seen in the C-33-A cell line, whereas high NNMT expression is found in the Ca-Ski, DoTc2, and SiHa EC cell lines. This is why we decided to use C-33-A, DoTc2, and SiHa cells to establish a range of NNMT expression in our cell models. Control cells were C-33-A/Vector, DoTc2/shNC, and SiHa/shNC, and we employed retroviral transduction to overexpress NNMT or an NNMT mutant (Y20) in C-33-A cells and to downregulate NNMT in DoTc2 and SiHa cells.

**Figure 2:**
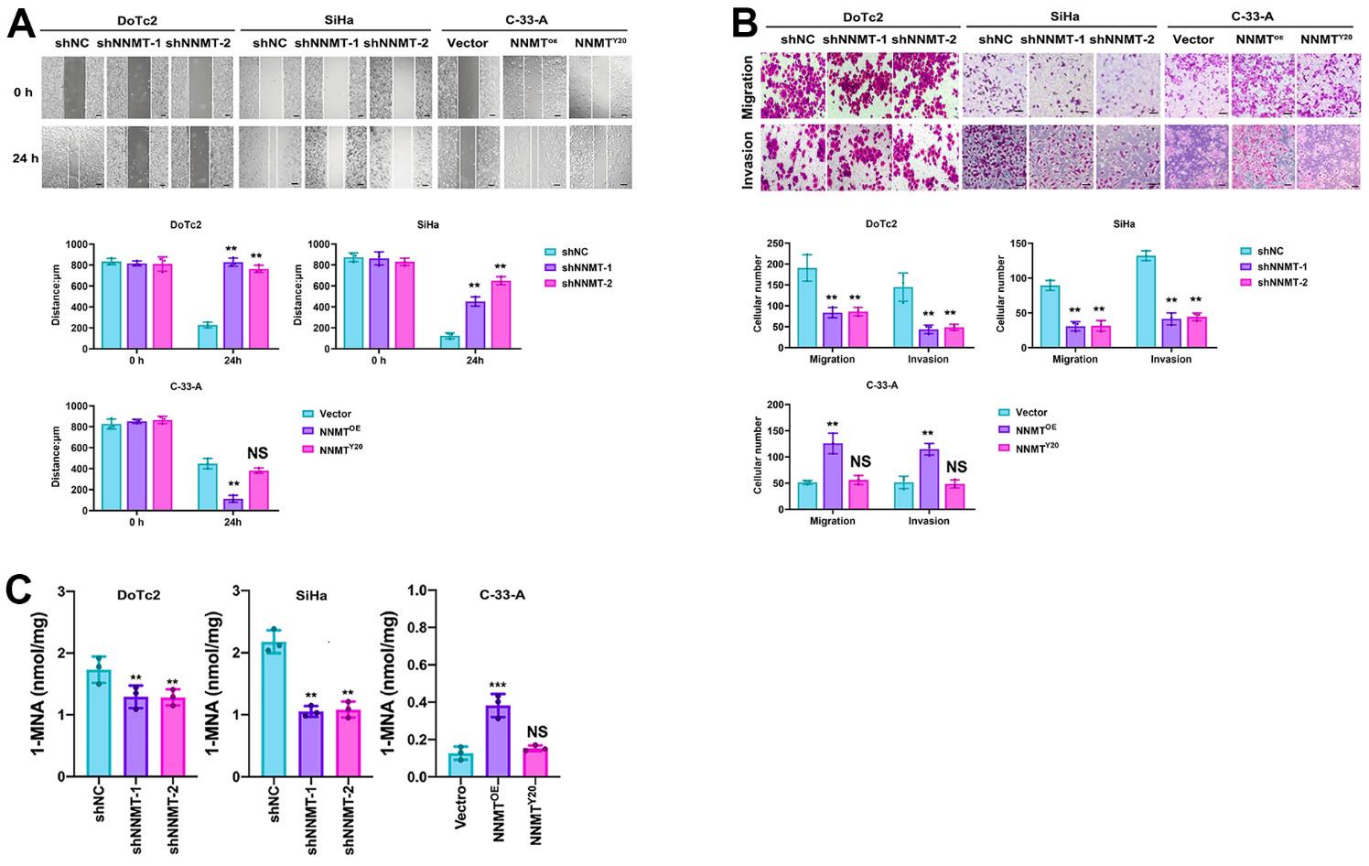
**NNMT promotes cell migration and invasion of EC cells.** (**A**) Results of wound healing assay (Scale: 100 μm). (**B**) Results of migration and invasion assays (Scale: 100 μm). (**C**) Intracellular 1-MNA concentrations using liquid chromatography-mass spectrometry. All experiments were conducted three times (**P < 0.01, ***P < 0.001, NS = Not significant).

The wound healing experiment demonstrated a substantial decrease in migration efficiency following NNMT downregulation in the DOTC2 and SIHA cell models. In contrast, an increase was seen following NNMT overexpression in the C-33-A cell models (Figure 2A). Similar results were seen in an assay measuring migration and invasion. Compared to when NNMT was overexpressed, the numbers of migrating and invading cells were drastically reduced when NNMT was downregulated (Figure 2B). These findings supported the hypothesis that NNMT enhances EC cell migration and invasion. SAM represents the methylation potential and serves as the methyl supply for several methyl catalytic processes within cells. The SAM level in DOTC2 and SIHA cells was raised by NNMT knockdown, whereas in C-33-A cells, it was lowered by NNMT overexpression. This finding demonstrated that NNMT’s catalytic activity depletes SAM, decreasing EC cells’ methylation capacity. One can only manufacture 1-MNA through the methyl transfer catalysis of NNMT, and the Y20 mutant is designed to render the NNMT protein incapable of catalysis. When compared to C-33-A/NNMTY20 and C-33-A/Vector, there was no discernible change in the levels of intracellular 1-MNA caused by NNMT downregulation or NNMT overexpression (Figure 2C). While overexpression of wild-type NNMT was shown to stimulate migration and invasion in EC (Figure 2A, 2B), the NNMTY20 mutant did not show this effect, suggesting that NNMT promotes migration and invasion in EC through its methyl transfer function.

### Reducing cholesterol levels and increasing membrane fluidity in EC cells are two ways NNMT facilitates cell migration and invasion

**Figure 3:**
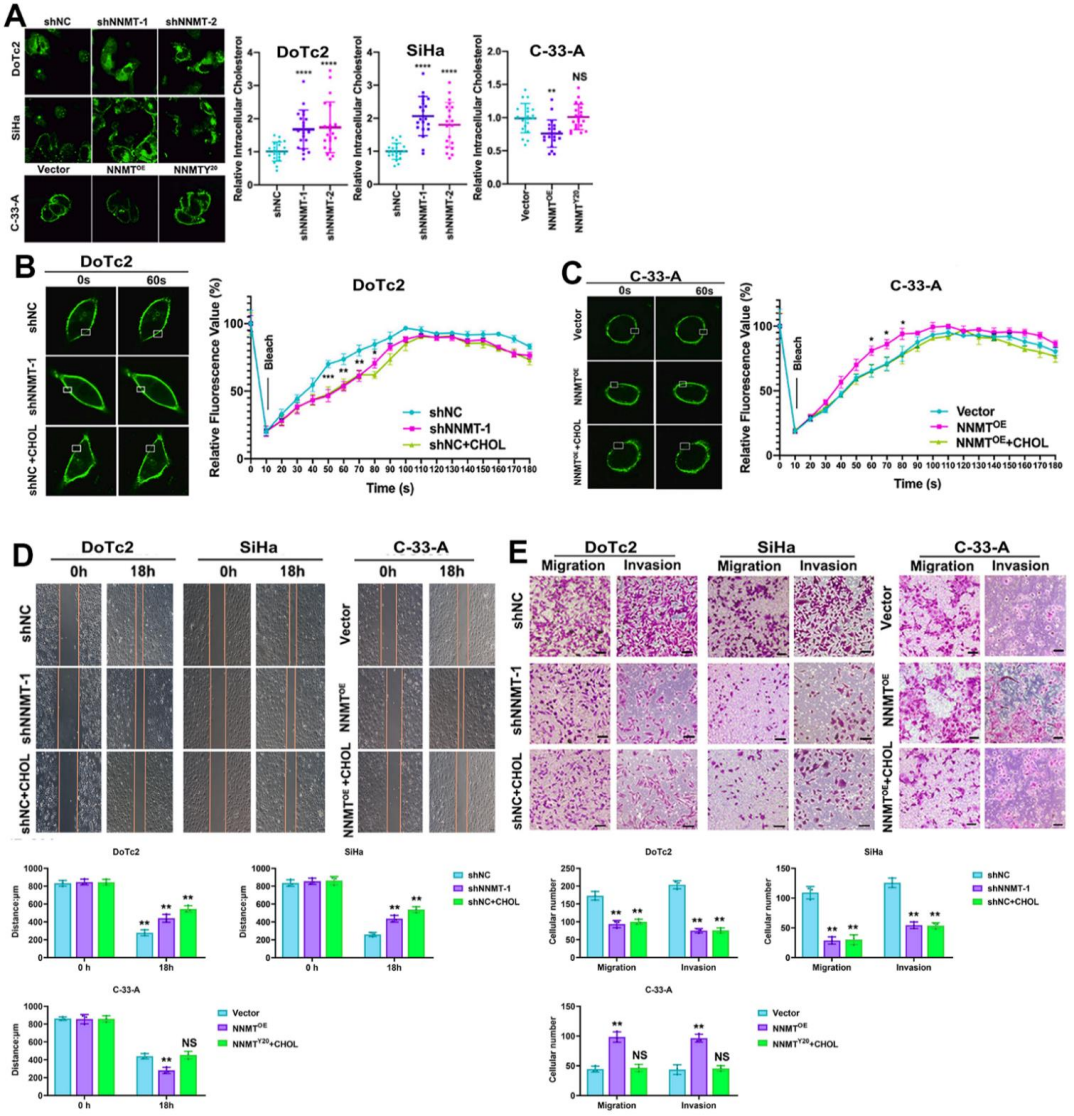
**NNMT promotes migration and invasion by reducing cholesterol in EC cells.** (**A**) Intracellular cholesterol levels detected by Amplex® Red cholesterol assay. (**B**, **C**) Representative results of Dil-C16 staining and fluorescence recovery recordings using confocal microscopy. (**D**) Representative results of cholesterol-treated wound healing assay. (**E**) Representative results of migration and invasion assays under cholesterol treatment (Scale: 100 μm). (*P < 0.05, **P < 0.01, ***P < 0.001, ****P < 0.0001, NS = not significant).

Cholesterol, a crucial structural component of cellular membranes that affects membrane fluidity and EMT, was analysed to provide light on the mechanism of NNMT on migration and invasion in EC cells. Amplex® green cholesterol test and filipin staining showed a substantial rise in intracellular cholesterol following NNMT downregulation. At the same time, both were negative after NNMT overexpression (Figure 3A). The filipin staining pattern of cholesterol on cell membranes followed the same pattern (Figure 3B). Intriguingly, increased NNMT expression had no effect on cholesterol in NNMTY20 mutants (Figure 3A, 3B), suggesting that NNMT reduces cholesterol in EC cells via its methyl transfer activity.

In addition, we could see the shift in membrane fluidity in our artificial cellular models. In contrast to C-33-A/NNMTOE cells, which recovered more quickly than C-33-A/Vector cells, the fluorescence in DoTc2 /shNNMT-1 cells recovered more slowly than in DoTc2 /shNC cells. In addition, cholesterol therapy considerably reduced fluorescence recovery (Figure 3B, 3C). These findings demonstrated that overexpressing NNMT in EC cells decreased their cholesterol levels, increasing their membrane mobility. Further evidence that NNMT expression improves cell migration and invasion by lowering cholesterol to increase membrane fluidity in EC cells was provided by the fact that cholesterol administration counteracted the beneficial effects of NNMT expression on migration and invasion (Figure 3D, 3E).

### Cholesterol levels in EC cells are lowered because NNMT upregulates ABCA1 expression, which increases cholesterol efflux

**Figure 4:**
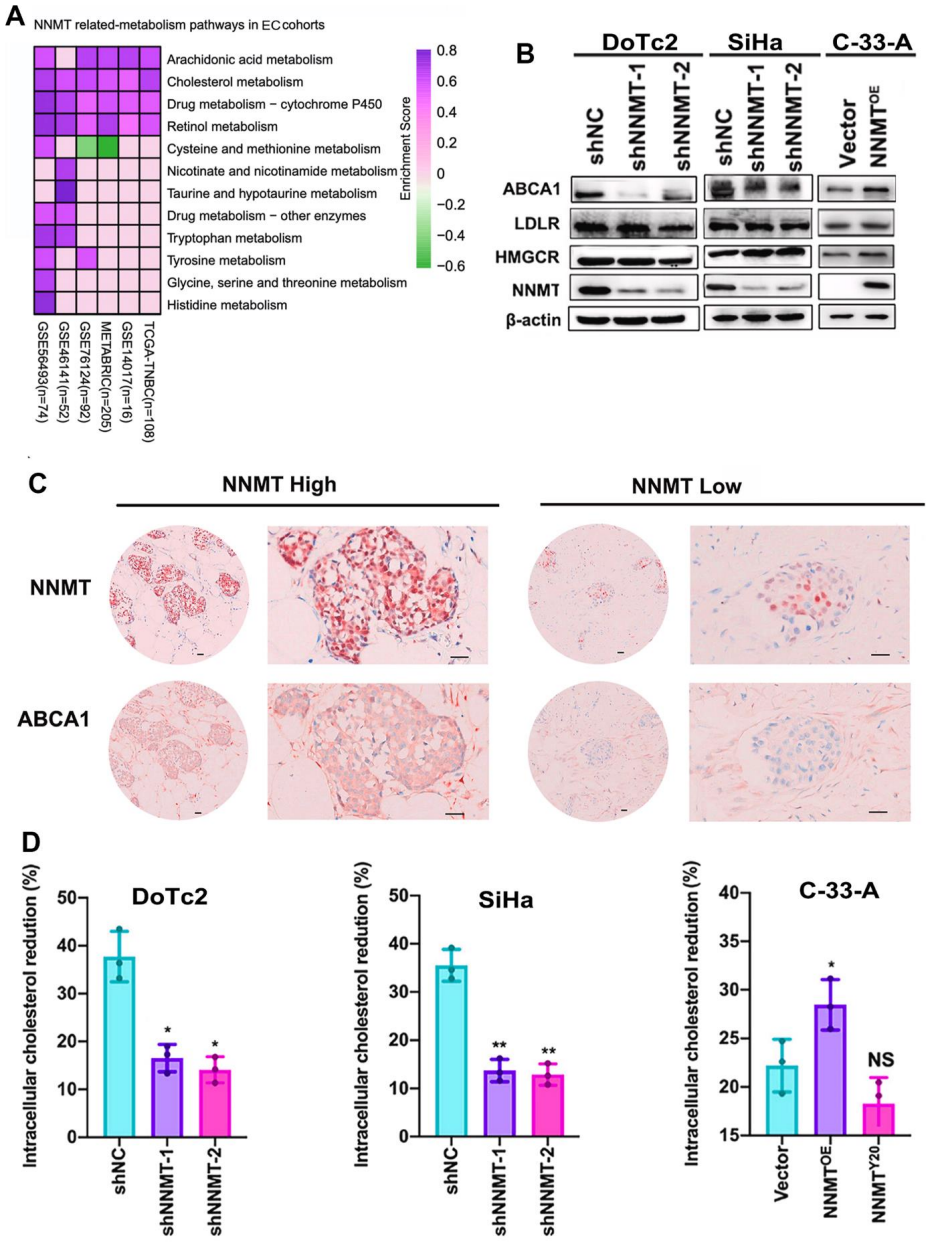
**Cholesterol efflux is improved thanks to NNMT’s ability to upregulate ABCA1.** (**A**) Gene set enrichment analysis (GSEA) was used to investigate pathways involved in NNMT-related metabolism. (**B**) Expression bands of proteins involved in cholesterol metabolism. (**C**) Example immunohistochemical staining for NNMT and ABCA1 proteins in EC tumors (Scale: 100 μm). (**D**) Inhibition of cholesterol production and uptake leads to a significant drop in intracellular cholesterol levels. (*P < 0.05, **P < 0.01, NS = not significant).

We used the TCGA gene set enrichment analysis (GSEA) to explore the possible connection between NNMT and cholesterol metabolism in EC. Cholesterol metabolism exhibited a higher enrichment score in all high NNMT mRNA expression groups compared to low NNMT mRNA expression groups (truncated by the median value) using gene set enrichment analysis (Figure 4A). Our cell models, DoTc2 and SiHa cells showed that downregulation of NNMT led to decreased levels of ABCA1 protein. At the same time, it did not affect HMGCR, LDLR, or mature SREBF2 protein. C-33-A cells with NNMT overexpression showed more significant levels of ABCA1 protein than HCC1937 control cells. Still, HMGCR, mature SREBF2, and LDLR proteins were not affected (Figure 4B). There is no discrepancy between the mRNA and protein results. There was a general trend toward changed ABCA1 expression at the mRNA and protein levels, which may indicate a relationship between NNMT expression and ABCA1 expression. Publicly available IHC data (Figure 4C) further supported the idea that NNMT and ABCA1 expressions are linked.

We measured intracellular cholesterol levels by blocking cholesterol biosynthesis and uptake to verify that NNMT lowers intracellular cholesterol via cholesterol efflux. Following a 24-hour incubation in complete media, cells were grown for 8 hours in a medium containing lipoprotein-deficient serum and the HMG-CoA reductase inhibitor Mevastatin. DoTc2/NC cells showed a more significant reduction in intracellular cholesterol than DoTc2/shNNMT-1 and DoTc2/shNNMT-2 cells (Figure 4D), whereas C-33-A/Vector cells showed a slower decline in intracellular cholesterol compared to C-33-A/NNMTOE cells. This finding suggests that NNMT decreases intracellular cholesterol by increasing cholesterol efflux. In light of these findings, it is clear that NNMT increases ABCA1 expression, increasing cholesterol efflux and reducing cholesterol levels in EC cells.

### ABCA1 expression is boosted by NNMT, which in turn lowers cholesterol levels and sets the stage for EMT

**Figure 5:**
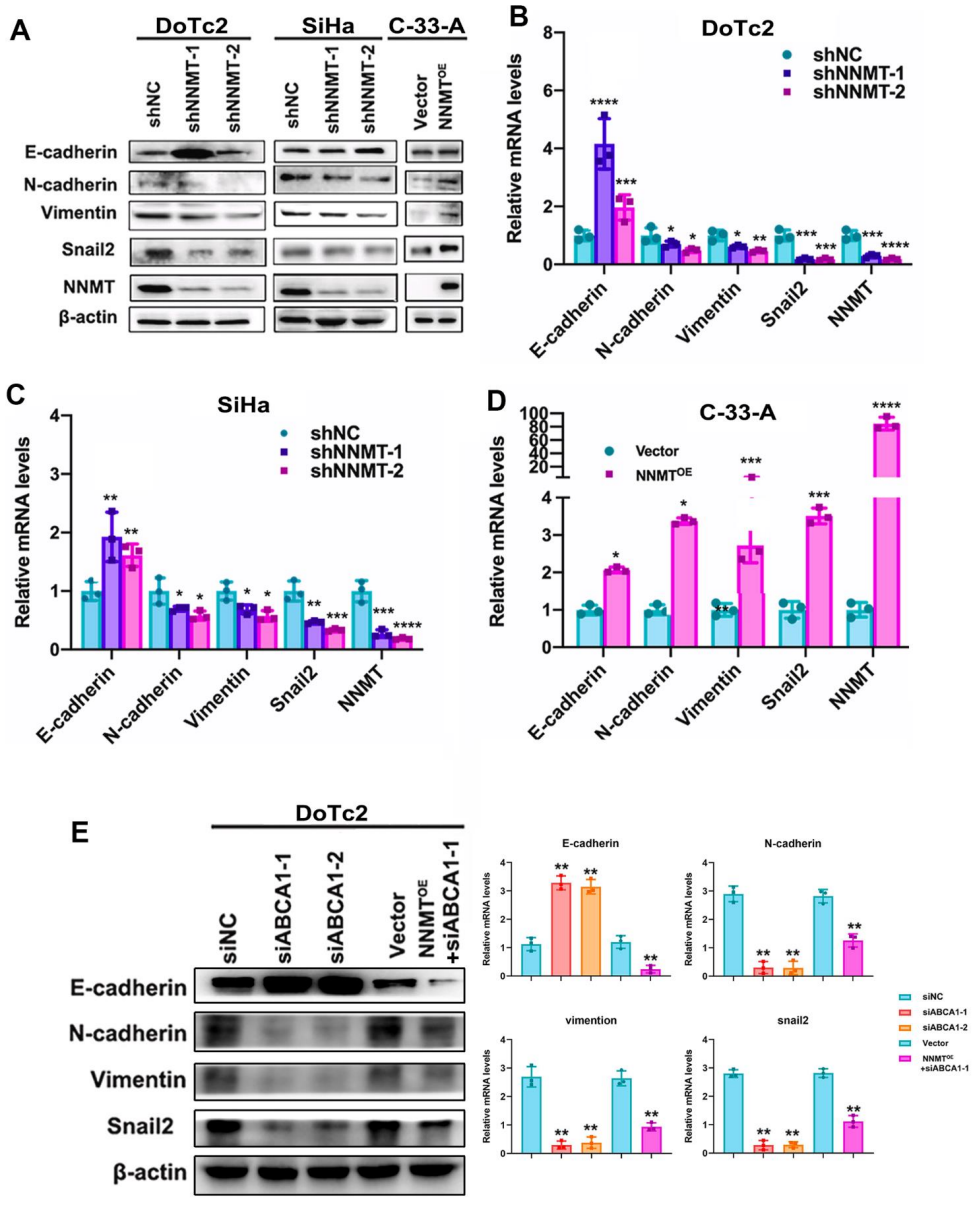
**Cholesterol is lowered thanks to ABCA1 expression being boosted by NNMT, triggering EMT in turn.** EMT-related protein western blotting result (**A**) as an example. Quantitative real-time polymerase chain reaction analysis of relative mRNA levels relating to the epigenetic regulatory transition (EMT) (**B**–**D**). (**E**) Western blotting and quantitative analysis of relative EMT-related protein levels in DoTc2 cells transfected with anti-ABCA1 siRNA; EMT-related protein levels are shown as a representative result. All experiments were conducted three times. The data are presented as a mean, standard error of the mean. (*P < 0.05, **P < 0.01, ***P < 0.001, ****P < 0.0001).

We observed a shift in molecules involved in EMT in our model cells, allowing us to deduce that NNMT reduces cholesterol. In contrast to the upregulation of the EMT marker E-cadherin (CDH1), the expression of the EMT marker N-cadherin (CDH2), vimentin, and the EMT transcription factor Snail2 was drastically reduced following NNMT downregulation. In contrast, after NNMT overexpression, N-cadherin, vimentin, and Snail2 proteins were markedly elevated, whereas E-cadherin protein was dramatically reduced (Figure 5A–5D). In addition, ABCA1 siRNA caused the same effect in DoTc2 cells (Figure 5E). The data presented here support the conclusion that NNMT stimulates ABCA1 expression, decreasing cholesterol and promoting EMT.

Using our own cohort data and publicly available statistics, we found that NNMT significantly correlates with metastasis and survival in EC. To add insult to injury, NNMT enhanced the activation of EMT, which improved the ability of cells to migrate, invade, and metastasize cells in the experiment. As a result, our results revealed that NNMT is a connection between cholesterol metabolism and metastasis in EC, providing a new molecular mechanism to explain the high metastasis of EC.

## DISCUSSION

Patients with EC often exhibit metabolic syndrome symptoms such as hyperlipidemia, hyperglycemia, hypertension, and other clinical manifestations [20, 21]. In an obese LKB1fl/fl p53fl/fl mouse model of EC, Guo et al. [22] found that metformin dramatically corrected the obesity-driven increase of lipid and protein production. Breast cancer [23], prostate cancer [24], and ectopic carcinoma [25] are only a few examples of obesity-related cancers in which ABCA1 has been shown to play a critical role in regulating metabolism. In addition, signalling pathways controlled by ABCA1 have emerged as essential players in controlling lipid metabolism in cancer. We were interested in this topic because our previous work using a high-throughput DNA/protein assay [25] showed that downregulation of ABCA1 expression in EC affected the translational factor activity of NNMT, suggesting that NNMT should interact with ABCA1 and be involved in EC lipid reprogramming and progression. Atherosclerosis [26], nonalcoholic fatty liver disease [27], cancer [28], and dementia [29] are only a few of the contexts in which a downregulation or absence of NNMT can visibly impact the cellular phenotype in a physiologically significant manner. In addition to its function in lipid catabolism and lysosomal biogenesis [14], NNMT is activated during hunger or caloric restriction.

For this reason, we jumped into a bioinformatics study of the TCGA data. Consistent with our hypothesis, both NNMT and ABCA1 were shown to be significantly linked to advanced disease at diagnosis (high FIGO stage) and shorter overall survival in EC. Additionally, the TCGA data of 543 EC patients revealed a strong correlation between NNMT and ABCA1 expression. Our TMA clinical data confirmed the findings of the bioinformatics study, showing that NNMT and ABCA1 were strongly correlated, and both were connected to the MI of EC. However, the precise interaction mechanism between NNMT and ABCA1 has yet to be elucidated. It was revealed in 2019 that NNMT drives PGC-1 expression in adipocytes to protect against diet-induced metabolic dysfunction [30, 31]. PGC-1 is one of the most critical coactivators of ABCA1. Further, we used ChIP assay and luciferase assay to verify that NNMT can bind to the promoter region of ABCA1 and control the expression and function of ABCA1 *in vitro*.

To make the stable metabolic product 1-MNA, NNMT transfers methyl groups from the cellular methyl donor SAM to NAM (the precursor of many molecules involved in energy metabolism, including NAD+ and NADH). Cancer cells’ abnormal behaviour, such as maintaining proliferative signalling, evading growth suppressors, and resisting cell death [2, 3], is essentially a product of NNMT’s control over methylation potential and energy metabolism. It has been revealed that NNMT is a prognostic marker for several malignancies [11] and promotes cell proliferation in many forms of cancer [32, 33]. To increase chemoresistance in EC, it has been reported that NNMT is highly expressed [21]. We recently discovered that NNMT is significantly more expressed in EC than in other cancers and plays a significant role in the metastasis and survival of EC patients using this extensive patient observation data and public datasets (TCGA). In addition, EC cell line C-33-A, which has a low metastasis capacity to begin with, benefits significantly from NNMT expression, both *in vitro* and *in vivo*. It appears to indicate that NNMT endows EC with solid metastatic potential. EC cell lines DoTc2 and SiHa, known for their metastasising ability, also express high levels of NNMT [34]. Consistent with these reports, our data showed that DoTc2 and SiHa cell lines with high NNMT expression had lower total intracellular cholesterol and SAM levels and higher 1-MNA levels, respectively, than the other cell lines with low or no NNMT expression. Additionally, DoTc2 and SiHa cell lines exhibited greater cell mobility, invasiveness, migration, c-Jun, and MEK/ERK signalling levels compared to the other cell lines, suggesting that NNMT played some general roles in EC metastasis.

Furthermore, we have first explored ABCA1 as a downstream signal through which NNMT increases UFA-GP accumulation during EC advancement by doing *in vitro* and *in vivo* lipidomics investigations. Lipidomic alterations or reprogramming may be significant in EC since Guo discovered large increases in lipid production and peroxidation in a genetically engineered mouse model of endometrioid adenocarcinoma [22]. The metastatic process is thought to be heavily influenced by EMT [35]. Metastatic potential is determined by the amount of cholesterol present in the cell membrane [23, 36], and increased membrane fluidity is a necessary cellular feature of this potential. We found that ECs with elevated NNMT levels had decreased cytoplasmic and membrane cholesterol levels due to increased expression of the ABC transporter protein ABCA1.

Our research also confirmed that elevated NNMT expression stimulates Snail2 and EMT. On the other hand, we discovered that downregulating Snail2 expression via ABCA1 can undo the EMT induced in EC due to overexpression of NNMT. Based on these findings, it appears that NNMT has a role in modulating Snail2 expression. Many different metabolic pathways, such as glucose, lipid, and cholesterol metabolism in the liver, and lipid metabolism in cancer cells [3, 19], were thought to be regulated by NNMT based on earlier research. Our research established a connection between NNMT’s essential methyl transfer function and its role in metabolism and EC function.

The protein CtBP, responsible for repressing cholesterol, has been shown by Zhao et al. [33] to stimulate TGF- signalling and EMT, promoting EC metastasis. Improved cell motility, EMT *in vitro*, and metastasis *in vivo* are all outcomes of elevated cholesterol efflux [23]. Our findings are similar to other studies showing that cholesterol can adversely influence EMT to boost cancer cells’ ability to metastasise. Our results prove that cholesterol is a barrier against cancer cell line-level EC metastasis. Several retrospective clinical investigations have investigated the association between cholesterol and EC metastatic risk, with most attention paid to blood cholesterol. Most of these studies found no correlation between cholesterol and metastasis, while others found a protective effect of cholesterol, yet others found cholesterol to be a significant risk factor [34]. As a result, there is some debate about whether or not a person’s cholesterol level affects their chance of developing EC metastases, and further study is needed.

## CONCLUSIONS

We discovered that NNMT decreases cytoplasmic and membrane cholesterol levels, which improves EC membrane fluidity and activates EMT via increased ABCA1 expression. Our findings reveal a novel molecular basis underlying EC’s high metastatic potential and raise the possibility that NNMT might be a future therapeutic target for this aggressive cancer.

## MATERIALS AND METHODS

### Cell lines and cell culture

The human EC cell lines Ca-Ski, DoTc2, SiHa, and C-33-A were purchased from American Type Culture Collection (ATCC, USA). All cells were cultured in DMEM/F12 medium (#A4192001, Thermo Fisher Scientific, USA), 1% antibiotic–antimycotic solution (#B120901, BasalMedia, Shanghai, China), and 10% fetal bovine serum (FBS) (#2275129, Gibco, Inchinnan, UK) at 37° C in a humidified 5% CO2 incubator.

### The characteristics of the patients and the immunohistochemistry analysis

The Human Research Ethics Committee at Guangzhou Medical University gave its clearance to proceed with this research (Permit Number: 20200702–009). Postoperative pathology findings at Guangzhou Women and Children’s Medical Center between January 1, 2018 and July 31, 2021 verified the diagnosis of EC in 309 patients. This span of time covers the period from January 1, 2018 to July 31, 2021 (Guangzhou, China). The clinical characteristics of these patients were extracted from their medical records. These characteristics included age and TNM stage (tumor diameter, lymph node metastasis, and distant metastasis). They were categorised according to the guidelines for EC developed by the Chinese Society of Clinical Oncology (Version 2020). These patients were followed up, and their overall survival (OS) was computed from the surgical treatment date to the date of their death or the date on which they had their most recent follow-up.

Tissue slices that had been formalin-fixed, paraffin-embedded, and cut to a thickness of 4 micrometres were deparaffinised, rehydrated, and stimulated with 0.01 M citrate buffer pH 6.0 before being microwave heated for thirty minutes. Afterwards, the sections were treated with 1% H_2_O_2_ for five minutes at room temperature, followed by normal goat serum for ten minutes at the same temperature. After that, the tissue sections were treated with a mouse monoclonal anti-human NNMT antibody (dilution 1:1400, #ab119758, Abcam, England) for 40 minutes, followed by incubation with a goat anti-mouse secondary antibody (#ab6789, Abcam, England) for 30 minutes. A DAB Chromogenic Kit (#AR1026, Boster Bio, China) was used to develop the sections of the slide. Two different pathologists, both of whom were unaware of the clinical details, carried out the analysis of the NNMT expression level. The proportion of positive cells and their corresponding intensity values were used to calculate the final score for the staining. Using the GraphPad Prism 9.0 software, a receiver operating characteristic (ROC) curve was constructed to determine the optimal cut-off value for the NNMT equation used in the calculation of EC.

### Bioinformatic analysis

The Uterine Corpus Endometrial Carcinoma (UCEC) data were downloaded from the Genomic Data Commons data portal, which can be accessed at https://portal.gdc.cancer.gov/. The Cancer Genome Atlas (TCGA) was used to collect this data. The dataset consisted of 543 EC specimens and 23 normal endometrial specimens; however, 9 repeated malignant cases were eliminated from the analysis. The Xena website at the University of California, Santa Cruz (UCSC) was used to get patient clinical information, gene-level copy number variation (CNV) profiles, gistic2 thresholds assessed using the GISTIC2.0 technique, and somatic nonsilent mutation (gene-level) data. The Database for Annotation, Visualization, and Integrated Discovery, or DAVID (version 6.8), has an extensive collection of functional annotation tools. These tools assist researchers in comprehending the biological significance of a vast list of genes. The cluster Profiler R program (version 1.3.1093) was used to show the Gene Ontology (GO) functional annotation findings and Kyoto Encyclopedia of Genes and Genomes (KEGG) analyses done on the proteins.

### Lentiviral infection and siRNA transfection

Cells were seeded at a density of 2 × 10^5^ cells per well in 12-well plates overnight. Then the cells were either co-cultured with lentivirus containing shRNA (Ribobio Co. Ltd, China) targeting the human NNMT gene or transfected with ABCA1 siRNA (20 nM), or control siRNA (20 nM, sc-37007). Further, cells were incubated in a refreshing medium.

### Constructed NNMT mutant and overexpression cell lines

Lipofectamine 3000 reagents were used to transfect HEK293T cells with 10 g of pLenti-Pur-NNMT (NCBI Reference Sequence: NM 00132045.1), pLenti-Pur-NNMT-Y20, or pLenti-Pur plasmid (VectorBuilder, China). After 48 hours, we collected the viral supernatant from the HEK 293T cells and filtered it using a 0.45 m filter (Millipore, USA). After 24 hours of incubation, the viral supernatant was discarded, and new media was added to the HCC1937 cells. Puromycin (1 M) was used to select cells for 5 days after infection.

### Wound healing assay

In 12-well tissue culture plates, cells were grown to 100% confluence before being placed in a starvation medium (0.1% FBS) for 12 hours. Cell monolayers were scraped using sterile 200 L pipette tips. Six sites along the cells’ migratory path were photographed at the start and end of the experiment (18 hours later) to obtain images of injured cells. Migratory efficacy was determined by comparing the wound gap at 0 h and 18 h post-wounding.

### Assay of invasion and migration

After 12 hours in low serum (0.1% FBS) medium, the cells were trypsinised and harvested. Also, in a 24-well plate, 2 × 10^4^ cells were resuspended in 200 L starvation media (0.1% FBS) and put into the upper chamber of Corning transparent Transwell 24-well permeable supports. Invasion assays were performed in the Matrigel (Corning, USA)-coated chamber, whereas migration assays were performed in the Matrigel-free chamber. There was an entire volume of growing media in the bottom chamber. Using a cotton swab, any cells that hadn’t migrated to the top of the chamber after 8 hours of incubation at 37 degrees Celsius and 5% carbon dioxide were eliminated. After 15 minutes in 100% methanol and two washes in PBS at room temperature, the migrating cells were fixed. Next, using a Carl Zeiss Microscope, 0.1% crystal violet dye was used to stain the cells before they were photographed at 40x magnification. The number of cells in 5 randomly chosen visual fields was recorded.

### Quantitation of cholesterol

Amplex® Green Cholesterol Assay (Thermo Fisher Scientific, USA) was used to measure total cellular cholesterol levels. Before adding the reaction mixture, the cells were lysed in 0.5% Triton X-100 and then incubated for 30 minutes while agitated (cholesterol oxidase, HRP). After a 30-minute dark incubation at 37 degrees Celsius, the samples were analysed with a fluorescence microplate reader set to 530 nm excitation and 590 nm emission detection.

### Filipin staining of cholesterol

4 × 10^4^ cells/well was the cell density used for growing in chambers. To prevent nonspecific binding, cells were washed with PBS, fixed with 4% paraformaldehyde for 15 minutes, and then blocked with 1% BSA in PBS for 1 hour at room temperature following treatment. The cells were stained for 2 hours at room temperature in the dark with 50 g/mL filipin after being rinsed twice with PBS. Using an excitatory wavelength of 405 nm and a Zeiss LSM 880 confocal microscope, we investigated the filipin signals (intracellular and on the cell membrane) of 20 randomly dyed cells. All images were taken with the same laser power, gain, and offset.

### RNA isolation and real-time quantitative PCR

Total RNA was isolated using TRIzol reagent (Invitrogen, USA) per the manufacturer’s instructions. The cDNA used for measuring mRNA levels was produced using a cDNA Synthesis kit (Thermo Fisher Scientific, USA). Table 1 details the sequences of all targeted primers. Using the universal® SYBR qPCR SuperMix Plus (Vazyme, China), qRT-PCR analysis was carried out in triplicate. The mRNA levels were normalised to that of GAPDH.

**Table 1:** The sequences of primers were used in the real time quantitative PCR.

**Gene**	**Forward sequence**	**Reverse sequence**
NNMT	GAATCAGGCTTCACCTCCAA	CCCAGGAGATTATGAAACACC
ABCA1	GCTGGATTTCTTGATCTGCTG	CTCTGTTCGGCTGAGCTACC
Twist1	GCAGCTATGTGGCTCACGA	TCTCTGGAAACAATGACATCTAGG
Vimentin	CGTCTCTGGCACGTCTTGA	GGGCATCCACTTCACAGGT
E-cadherin	CAGTCAAAAGGCCTCTACGG	GGCAGCTGATGGGAGGAATA
N-cadherin	GCTGCCACTGTGATGATGTC	GGACAGTTCCTGAGGGATCA
β-actin	CATGTACGTTGCTATCCAGGC	CTCCTTAATGTCACGCACGAT

### Western blot analysis

We first rinsed the cells in cold PBS before homogenising them in RIPA buffer (Solarbio, China) containing protease inhibitors (Boster, China) and lysing them at 4 degrees Celsius for 30 minutes before centrifuging them at 12000 revolutions per minute for 15 minutes. Preparation of the nuclear lysate for c-Jun protein detection was performed with the Nuclear Extract Kit (Abcam, England). BCA Protein Assay Kits were used to determine protein concentration (Solarbio, China). After separating the proteins on an SDS- polyacrylamide gel, the gels were transferred to a 5% milk in a TBST-blocked Immobilon P Transfer Membrane (Millipore, USA) for 1 hour at room temperature. After washing the membrane in Tris-buffered saline containing Tween 20, it was treated with a horseradish peroxidase-conjugated secondary antibody for 1 hour at room temperature after overnight incubation with the primary antibody at 4 degrees Celsius. After being washed in TBST, the chemiluminescence detection reagents were used to view the signals, which were then photographed using a ChemiDoc Touch Imaging system and processed with Image lab software (Tanon, China). The anti-GAPDH (ab181602), anti-NNMT (ab119758) antibody, EMT Marker / Epithelial to Mesenchymal Transition Marker Panel (ab216833) and goat anti-mouse secondary antibody (ab6789) were all purchased from Abcam (England).

### Quantification of 1-MNA and SAM by LC-MS/MS

The AB SCIEX Triple QuadTM 4500MD mass spectrometry equipment was used for the LC-MS/MS analysis of SAM and 1-MNA. A 1% zinc sulfate solution containing a stable isotope labelled internal standard (N-MNA-d4) was diluted to 250 L and added to 50 L of cell samples or a series of concentration standards (1-MNA and SAM). To prepare samples for LC-MS/MS analysis, they were shaken at 400 rpm for 30 minutes before being centrifuged at 14000 rpm for 20 minutes. To separate the material by liquid chromatography, 5 L was injected into the Eclipse XDB-C18 column (4.6 × 150 mm, 5 m; Agilent) attached to the JasperTM (SCIEX) LC system. Before being pumped into the mass spectrometer’s electrospray ionisation (ESI) chamber at a rate of 1 mL/min, the isocratic mobile phase, a 0.1% formic acid and methanol combination (v/v), was filtered through a 0.22 m membrane filter (Millipore, USA) and degassed ultrasonically for 15 minutes. 1-MNA was retained for 1.31 min, whereas SAM and N-MNA-d4 were retained for 1.20 and 1.65 min. The concentration is ultimately measured in terms of the cell’s protein content.

### Membrane fluidity assay by fluorescence recovery after photobleaching (FRAP)

After 12 hours of growth in a Lab-TEK II chamber slide (Thermo Fisher Scientific, USA), cells were incubated in 1 g/mL Dil-C16 solution for 60 seconds at room temperature. The imaging chamber of the Zeiss LSM 880 confocal microscope was preheated to 37 degrees Celsius, and the chamber slide was placed inside. A spot of membrane measuring just 1 μm was photobleached using a 63 oil objective and a 100% laser (561 nm). Every 10 seconds for 3 minutes, the fluorescence intensity before and after bleaching was recorded. By subtracting the background values from the measured fluorescence intensities at each time point, we were able to eliminate experimental noise and produce a smooth recovery curve for the fluorescence signal.

### Statistical analysis

SPSS 25.0 (IBM, USA) was used for the statistical analysis. Pearson’s χ^2^ test was used to examine the associations between NNMT expression and clinicopathological features in our clinical data. The Kaplan-Meier (KM) survival analysis technique was used, and the log-rank test was used for statistical comparisons. Two-tailed Student’s t-tests were used to determine whether or not there was a statistically significant difference between groups *in vitro* and *in vivo* studies. Three separate laboratories conducted the *in vitro* tests. The levels of statistical significance are as follows: *P < 0.05, **P < 0.01, ***P < 0.001, ****P < 0.0001.

### Availability of data and material

All datasets used and analysed during the current study are available from the corresponding author upon reasonable request.
